# Sociotechnical Challenges of Digital Health in Nursing Practice During the COVID-19 Pandemic: National Study

**DOI:** 10.2196/46819

**Published:** 2023-08-16

**Authors:** Karen Livesay, Sacha Petersen, Ruby Walter, Lin Zhao, Kerryn Butler-Henderson, Robab Abdolkhani

**Affiliations:** 1 School of Health and Biomedical Sciences Science, Technology, Engineering, and Mathematics College Royal Melbourne Institute of Technology University Melbourne Australia

**Keywords:** nursing informatics, digital health, COVID-19 pandemic, workforce, sociotechnical approach

## Abstract

**Background:**

The COVID-19 pandemic has accelerated the use of digital health innovations, which has greatly impacted nursing practice. However, little is known about the use of digital health services by nurses and how this has changed during the pandemic.

**Objective:**

This study explored the sociotechnical challenges that nurses encountered in using digital health services implemented during the pandemic and, accordingly, what digital health capabilities they expect from the emerging workforce.

**Methods:**

Five groups of nurses, including chief nursing information officers, nurses, clinical educators, nurse representatives at digital health vendor companies, and nurse representatives in government bodies across Australia were interviewed. They were asked about their experience of digital health during the pandemic, their sociotechnical challenges, and their expectations of the digital health capabilities of emerging nurses to overcome these challenges. Interviews were deductively analyzed based on 8 sociotechnical themes, including technical challenges, nurse-technology interaction, clinical content management, training and human resources, communication and workflow, internal policies and guidelines, external factors, and effectiveness assessment of digital health for postpandemic use.

**Results:**

Sixteen participants were interviewed. Human factors and clinical workflow challenges were highly mentioned. Nurses’ lack of knowledge and involvement in digital health implementation and evaluation led to inefficient use of these technologies during the pandemic. They expected the emerging workforce to be digitally literate and actively engaged in digital health interventions beyond documentation, such as data analytics and decision-making.

**Conclusions:**

Nurses should be involved in digital health interventions to efficiently use these technologies and provide safe and quality care. Collaborative efforts among policy makers, vendors, and clinical and academic industries can leverage digital health capabilities in the nursing workforce.

## Introduction

### Background

Health care delivery has greatly changed with the growing adoption of digital health technologies. These changes have impacted nursing practice, as nurses are the largest group of health care providers, representing more than 59% of the world’s health care workforce. The World Health Organization has recognized digital health as a critical catalyst to advance universal health coverage. Therefore, embedding digital health in nursing practice can significantly advance the services and improve health outcomes [1]. Digital health use in nursing practice has shown significant benefits at the personal and organizational levels, such as improved efficiency in service delivery [2], increased organizational support, and better communication between professionals and patients [3]. However, these are only possible if nurses are equipped with digital health skills and are actively involved in the design and implementation processes. At the frontline, nurses are the connection points between patients and digital health technologies. However, despite this vital communication role, the involvement of nurses in digital health is often low. The key challenges in the efficient use of digital health in nursing are the lack of basic skills and the lack of access to digital health technologies. Although digital health competencies for nurses have been developed by related national and international institutions [4-6], they are not yet formally integrated into nursing practice and education.

The COVID-19 pandemic has accelerated the use of digital health innovations, such as virtual consultations, remote patient monitoring, and digital transformation, associated with the speed of change in providing treatment and care during the pandemic [7]. Nurses were significantly impacted by digital transformation [8,9].

The need for digital health skills in nursing significantly increased after the COVID-19 outbreak [10]. Our previous study acknowledged the lack of global awareness about the skills required by nurses during nursing practice and education to efficiently act in digitally enabled care [11]. Australia’s response to the COVID-19 pandemic included rapid implementation of digital health, namely telehealth, in both primary and acute care settings. In 2021, 950,700 telehealth visits were estimated to have been conducted for people aged 55 to 64 years across Australia [12]. The Australian Government provided principles for digital health to ensure continuous high-quality care [13]. However, at the time of conducting this study, it was unclear to what extent these principles were executed in nursing practice and what challenges nurses faced in using the rapidly deployed technologies.

Exploration of digital health use in nursing practice requires consideration that the design and performance of any organizational system can only be understood and improved if both “social” and “technical” aspects are brought together and treated as interdependent parts of a complex system [14]. Nowadays, health care organizations are profoundly affected by technological progress, and a flexible customized change model is required to fit the social network of the specific organization into which technology is being introduced [15].

### Significance and Objective

Since the beginning of the pandemic, a large amount of literature has discussed its impact on nursing care, such as the interruption in workflow [16-19], the pressure and mental health issues among nurses [20-26], and the requirement of training [27,28]. Moreover, digital health implementations and their implications for nursing practice, such as telehealth adoption, informatics-related competencies, and the shift to virtual training for nursing students, have been studied [9,11,29]. However, one missing key theme is the processes in which interactions between nurses and technologies occur within the complex health care environment. There is limited clarity regarding the digital health technologies established during the pandemic, the communication between different tools and users, the workflow and processes that impact the data and information produced and the decisions made, the availability of human and financial resources and training to support the workflow, and the policies and regulations in place to inform efficient implementation and use of technologies, as outlined in our previous study [11]. It is essential to comprehend the human, technology, and process-related aspects of these interactions that impact the nursing practice, and it will be evident which competency requirements are needed to meet sociotechnical challenges.

Another missing aspect is knowing the sociotechnical challenges in the digital health pipeline from design to development, implementation, and evaluation, which can impact nursing practice [30]. With the pressure on nursing practice brought by the pandemic, there has been limited time to study nurses’ engagement in the design and development of technologies, and even before the pandemic, digital health interventions were only studied at the implementation and utilization stages, with a lack of assessment of usability for integrating technologies into routine clinical care [31,32]. Therefore, there is a lack of user insights and feedback, and thus, it is difficult for developers to establish appropriate strategies for technology development that meet real-world nursing needs.

This study addresses the sociotechnical challenges in nursing practice associated with the digital health transformation during the COVID-19 pandemic, as well as nurses’ expectations of digital health competencies in the emerging workforce for providing safe and quality patient care.

## Methods

### Study Design

The research team comprises digital health and nurse researchers. This national study follows the qualitative research principles suggested for digital health studies [33]. A semistructured interview protocol was developed and pretested with external experts, including academic, clinical, and policy-maker nurses, to refine the protocol before conducting the primary interviews. The protocol contained the following parts:

- Part 1: This part included 3 questions about nurses’ roles and years of experience in their current setting and the types of digital health technologies they used during the COVID-19 pandemic, followed by 8 questions concerning 8 sociotechnical aspects, including technical issues related to the hardware, software, and network; nurse-computer interface; content management; training and human resources; communication and workflow; internal policies and guidelines; external factors; and effectiveness assessment for postpandemic use and integration.
- Part 2: This part included 4 questions about the expectations of new graduates regarding digital health competencies to overcome challenges and efficiently apply digital health in nursing practice. The questions were related to awareness, theoretical knowledge, practical skills, and competencies for digital health sustainability and integration into practice in the emerging nursing workforce.

Interviews enable researchers to explore a phenomenon in depth through the interviewee’s experiences, opinions, and insights [34]. The research topic, study design, and interview protocol development were based on the findings and implications from our previous literature review [11]. The findings reported in the literature review were not necessarily based on nurses’ experiences across the entire digital health pipeline or collected from different nurses involved in various stages of the pipeline. This interview study obtained information from various nurses involved in digital health within and outside health care settings regarding the sociotechnical challenges of digital health in nursing practice.

The ethics application and call for participation were undertaken from February to April 2022. The participant recruitment process, data collection, transcription, and analysis were conducted from May to December 2022.

### Ethics Approval

This research received human ethics approval from Royal Melbourne Institute of Technology University (ID: 25054), and it is part of a larger translational project that will provide directions for the next stage.

### Participant Recruitment

Nurses involved in digital health design, development, implementation, use, evaluation, regulation, and policy making were approached to participate in order to gain a comprehensive understanding of the sociotechnical issues related to the use of digital health in nursing practice. The participant groups included the following types of nurses across Australia:

- Nurses in clinical settings, including chief nursing information officers, clinical educators, and nurses within health care settings who had experienced the use of digital health initiatives for patient care. Participants in this group were labeled with code “C.”
- Nurse representatives in digital health vendor companies that partnered with health care settings for digital health implementation. Participants in this group were labeled with code “V.”
- Nurse representatives in the government who contributed to policy making for digital health implementation in health care settings. Participants in this group were labeled with code “P.”

A call for participation was circulated through the researchers’ professional networks in nursing and digital health communities in the country and on social media platforms. Moreover, a snowball sampling technique was used to recruit further participants. The aim was to include up to five participants from each of the 3 clinical nurse categories in the first group and up to three participants in the other 2 groups. Despite multiple calls for participants, no further individuals indicated a desire to participate. As we noted repetition and similarities in participant responses and included a cross-section of geographical locations and nursing contexts, we ceased recruitment. Generalization was not an aim of this research, but adequate data were obtained to answer the research questions through rich and nuanced responses.

### Data Collection

Potential participants who expressed interest in participating were contacted to confirm that they met the study inclusion criteria. They were given a participant information sheet and a consent form to sign before the interview. One researcher arranged a date and time for the interview at each participant’s convenience.

At each interview, the study purpose, study scope, research questions, and interview structure were briefly explained before asking the interview questions. To ensure consistency, 2 researchers conducted all the interviews via the Microsoft Teams platform (Microsoft Corp). Each interview took between 30 minutes and 1 hour. Interviews were audio recorded and transcribed verbatim.

### Data Analysis

The interview transcripts were thematically coded and analyzed manually [35]. Thematic descriptions were coded deductively according to the parts of the interview protocol and analyzed in the following three core categories:

1. Types of digital health services implemented during the pandemic in primary and tertiary care settings.
2. Sociotechnical challenges identified in using digital health in nursing practice. The 8-dimensional sociotechnical model designed by Sitting and Singh [15] was selected to deductively analyze the interviews and explore the interactions between digital health technologies and nurses. This approach was applied to better understand the nurses’ experiences of issues inherent in the design, development, implementation, use, and evaluation of digital health solutions during the pandemic. The findings were synthesized based on the 8 themes of the sociotechnical approach, including technical issues related to the hardware, software, and network; nurse-computer interface; content management; training and human resources; communication and workflow; internal policies and guidelines; external factors; and effectiveness assessment for postpandemic use and integration.
3. Competency requirements to efficiently apply digital health in nursing practice.

The findings were then reviewed by the research team and discussed in multiple meetings to reach an agreement on coding.

## Results

### Characteristics of Interview Participants

Sixteen individuals participated in the interview (Table 1). Most participants were in the clinical nursing group. They were distributed across 2 primary care settings and 10 hospitals nationwide.

**Table 1:** Characteristics of the interview participants.

Participant group	Number	State	Participant ID
Chief nursing information officers	3	New South Wales and Victoria	C1, C2, and C3
Nurses	6	Queensland, New South Wales, Tasmania, and Victoria	C4, C5, C6, C7, C8, and C9
Clinical educators	3	Northern Territory and Victoria	C10, C11, and C12
Nurse representatives in digital health vendor companies	2	Queensland and South Australia	V1 and V2
Nurse representatives in government bodies	2	Queensland and Tasmania	P1 and P2

### Types of Digital Health Services Implemented or Expanded During the Pandemic

The pandemic shifted face-to-face appointments to virtual appointments using various technologies. The digital health services mentioned by participants were virtual care, telehealth, telemonitoring, remote patient monitoring, and care at home. These services were either applied to monitor COVID-19 symptoms or to monitor other health conditions remotely. The technologies used in these services included various teleconference platforms, such as Zoom, Microsoft Teams, and Skype; mobile apps; wearable devices; and chatbots.

Health care systems had an urgent need for software systems that could help screen the high volume of patients before hospital admission. Telehealth services enhanced access to care across the country during the pandemic, as outlined by participants V1 and P2.

However, the newness of the technologies used in telehealth and the high load of data produced, which were not integrated with electronic medical records (EMRs), were challenging for nurses.

There is probably a cognitive load that started to increase, although those software systems might have improved the efficiency of looking at those the patient in different areas. It's starting to be difficult for some nurses to be able to follow and manage different software at the same time and most of the software is quite new, especially in those last two years, and did not yet integrate to the EMR.

Participant V1

There was a disproportionate balance in confronting the pandemic between health care settings that were digitally equipped and those that were not or were at the beginning of digital health adoption. For example, training of nurses was more challenging in settings that had not implemented digital health before, as mentioned by participant P2. In health care settings that had implemented digital health and EMRs for many years, more advanced applications were implemented during the pandemic to augment the existing systems. For example, participant C2 mentioned that mobile devices were provided for nurses to access EMRs from anywhere across the hospital.

### Sociotechnical Challenges of Digital Health in Nursing Practice During the COVID-19 Pandemic

The interviewees addressed the sociotechnical challenges they experienced in digital health adoption and use during the pandemic. These challenges are outlined in Table 2.

**Table 2:** Participants’ perspectives on the sociotechnical challenges regarding digital health in nursing practice during the pandemic.

Sociotechnical aspects	Challenges (participant ID^a^)
Technical challenges	Lack of internet connectivity in distant areas (P1, P2, C7, and C8) Interoperability challenges among various devices (P1, C2, and C12) Inability to troubleshoot devices (C1, C7, and C8) Difficulties in the infrastructure network (C1, C7, C10, and C12) Difficulties in reporting errors (C4 and C6)
Nurse-technology interaction	Challenging user interface for immediate clinical actions (P1 and C5) Fear and demotivation in interacting and using a new technology due to lack of preparedness (V2) Heavy load of digital documentation and nurse shortage (C3, C6, C8, and C9) Interaction with various screens in telehealth consultations is overwhelming (C5, C7, and C11)
Content management	Inability of digital health systems to store and analyze a large volume of collected data (C1 and C12) Lack of time to manage the digital content for quality assurance (C12) Lack of access to and use of patient-reported outcome measures to improve remote management (C11)
Training/human resources	Lack of digital health literacy in the senior nursing workforce (P1, C4, C5, C8, and C9) Lack of consistent and continuous formal training (P1, C3, C8, C9, and C11) Lack of time for appropriate training (P1) More cumbersome training in settings that were new to digital health (P2) New technologies led to the emergence of new roles for nurses that required new skillsets (C1) Lack of chief nursing informatics officer roles (P1) Lack of the use of the informatics workforce in technology implementations (C3 and C4) Lack of economists’ perspectives in digital health business models (C4)
Communication and workflow	Difficulties in data collection from siloed technologies that are not integrated into the electronic medical records (V1, C9, and C10) Lack of effective communication among nurses and other stakeholders in using digital health (V1, C5, C6, C7, C10, and C11) Lack of communication between managers and ward nurses to understand nurse-specific needs in using digital health (V2) Lack of nurses’ involvement in critical decision-making in digital health implementation (C3, C4, C7, and C10) Interruptions in nurses’ workflows due to lack of computers at the bedside (C4) Difficulty in communication between nurses and patients in using mobile apps (C8) Challenges in using interpreters in virtual appointments (C8)
Internal policies and guidelines	Current legislations are not applicable nationwide (P1 and C7) Lack of an organizational approach to identify the practice problems that can be solved by a particular technology (V1) Lack of strategies on how to improve access to virtual care for communities with culturally and linguistically diverse backgrounds (C1)
External factors	Lack of legislation to support data transfer between primary and acute care settings (P2) Lack of involvement of external experts in using digital health technologies (C4)
Effectiveness assessment for postpandemic use and integration	Lack of nurse’ evaluation of the implemented digital health services (P1 and C12) Lack of funding for continuous evaluation (P1) Lack of workforce to know and conduct the evaluation (C1) Lack of feedback and measurement of nurse performance in digital health systems (C12)

^a^The participant IDs are clarified in Table 1.

#### Technical Challenges

One of the major technical barriers was lack of internet connectivity in remote areas to enable data exchange and communication in virtual consultations. One of the policy makers (participant P1) and a chief nursing informatics officer (participant C1) mentioned that the current infrastructure was a large barrier to efficient communication.

We only have two fiber cables that run between us and the mainland, and interestingly both were cut through recently, so we had no IT on the island, which again shows our vulnerability in this space.

Participant P1

I think what lets us down sometimes is our infrastructure network in Australia. It lets us down significantly when we're trying to do virtual care and telehealth.

Participant C1

Additionally, participants from 2 groups of clinical nursing professionals (participants C1, C4, C6, C7, and C8) mentioned difficulties in troubleshooting and reporting errors.

#### Interaction With Technologies

Most of the nursing workforce was not literate enough to use technologies efficiently under emergency circumstances. Moreover, there were challenges related to the design and functionality of digital health technologies, which were not responsive to emergency clinical demands and led to inappropriate use of clinical information.

Whatever the product happens to be, on a functional level, is this going to work day to day? No, because we've got 20 clicks to go through and five pages, and it's not workable or you've missed a key element of information that we absolutely need to capture. But there's no way for us to put that in a meaningful way.

Participant P1

Health care settings faced a nursing workforce shortage, while there was a high need for digital documentation of the large volume of collected data. Some participants (C3, C6, C8, and C9) reported that data were duplicated and repeatedly executed and stored in various places in the EMR.

The key changes around clinical information systems is that you should be documenting once and that documentation is pulled into various places rather than nurses being the workaround to capture that information.

Participant C8

#### Content Management

Various tools collected large amounts of data at the peak of the pandemic, which was overwhelming to nurses, as the current systems did not have the capacity to manage the data appropriately.

We had hundreds of patients needing care by nurses, and we and they were overwhelmed by the data that was coming through and the inability to be able to monitor all of the data in a safe way. And some of the challenges we had with our digital systems not being bespoke enough to manage that with the clinical decision support and the alerting in place.

Participant C1

Participant C12 was concerned that most of the nurses’ time in using digital health tools was spent on documentation, which left no time for quality assessment or analysis and interpretation.

We'd spent much time at the end of our shift writing our notes, but we never looked at the content and the quality and that value, what value does it add? Why am I writing that?

Participant C12

Moreover, according to participant C11, the use of patient-reported outcomes was lacking, which, if collected efficiently, would help in having a more comprehensive picture of the patient status.

#### Training/Human Resources

The pandemic emphasized the need for continuous immediate training in digital health beyond the usual training cycles, which requires established plans to support the workforce for prompt changes.

Knowing that we have a workforce that can just continually keep pivoting as and when they need to, and they don't get changed fatigue because they're constantly having to relearn. And so this is where I think having dedicated rules in the health system are of benefit to support that work that's going through and can support across the workforce so that you don't feel vulnerable.

Participant P1

However, the same participant noticed that none of the digital health implementation failures was directly due to a lack of workforce competencies.

So I haven't heard that we've tried to implement something, and we haven't been able to do it because the end user hasn't had the skills to adopt it, and we've had to basically scrap it.

Participant P1

A clinical educator (participant C10) said that as most digital health initiatives implemented during the pandemic were still research-based projects, it was a waste of time to allow nurses to participate in such initiatives rather than clinical practice, due to staff shortages.

It jeopardizes our workforce supply. I called a number of times for project nurses to be called back to the clinical setting because I felt that it was not a good use of their time at this time.

Participant C10

Participant C9 was concerned that even some specialists are unfamiliar with the basics of IT and digital literacy. There was a worry about how these specialists will cope with the digital transformation. Participant P2 mentioned that most of the training for nurses on using digital health is conducted internally in health care settings and not through vendors or by following external digital health policies.

Owing to high demand for technologies and lack of time for providing real individual or group training, digital health vendors moved toward self-help training modules. They provided types of escalation sessions in which users can choose to have individual training (participant V1). This would help enhance autonomy at work and less reliance on the IT team.

In terms of the mode of training, participant C1 suggested the blended approach to respond to nurses with different need levels.

Participant C3 indicated that the health and nursing informatics teams were not actively involved in digital transformation during the COVID-19 pandemic owing to time pressure.

The team that led the work for COVID and the transformation in that space didn't leverage the informatics team. Primarily because it was just so fast-moving that it was done with our transformation team.

Participant C3

The emergence of new technologies defined new roles and responsibilities for nurses in virtual care and telehealth, such as a digital health coach who provides training to other nurses (participant C1). However, some health care settings did not have the infrastructure to adopt the technologies. Moreover, the nursing workforce might feel unsafe and demotivated when new technology is rolled out.

Nurses won't be interested. They will not feel safe at work. They won't come to work. it's really hard. It's 12 months, if not more, to rebuild that relationship, to come in and train them and promise them that it will work.

Participant V2

#### Communication and Workflow

During the pandemic, there was a massive shift from paper-based workflow to digital forms and activities (participant C1). Health care systems increasingly relied on technologies to help in responding to the pandemic. Technology vendors said the newness of the tools, the high volume of data generated, and the lack of capability among the nursing workforce to use the tools efficiently were challenging.

There is no capability to actually have a contingency plan with those tools are failing because they don't have the workforce, they don't have the time to do it.

Participant V1

Nurses have the natural skill to communicate with other stakeholders to provide better care outcomes. However, they were not fully involved in digital health implementation due to a lack of analytical and critical approaches for the technical interface, as mentioned by participant V1. Moreover, based on the note by participant C5, it is difficult to communicate with the technical team when accessing virtual care services. There was also a lack of appropriate continuous communication between nurse managers and the nurses within different wards, which led to a different understanding of needs and challenges in using various technologies, as mentioned by participant V2.

The use of siloed technologies that were not integrated led to repeated tasks and duplication, which was time consuming, as mentioned by participant C9.

#### Internal and External Policies, Guidelines, and Legislation

Current policies did not keep pace with the fast changes of digital transformation, as highlighted by one of the policy makers.

Our existing legislation is not responsive to the technology pace. So I think this is something that we're still grappling with because the legislation doesn't change quickly.

Participant P1

One challenge for digital health vendors was that nurses often do not have a comprehensive organization-wide understanding of how a particular technology improves the workflow in different departments of a care setting.

Most of the time, a lot of nurses are interested in our platform and actually come to us to know more about it. The difficulty here is always about how those nurses can bring this perspective to their organization. Sometimes it's very difficult to understand what is the real problem on the floor. I think the real perspective from the nursing that is sometimes missing in a digital health space is for them to be able to bring to their executive like, these are the problems we need to be solving. And most of the discussions are very high level or very often projects are unsuccessful and not going forward because the executives lack the perspective of what's in it for the clinicians.

Participant V1

Another access challenge was raised in regard to virtual care services for people from culturally and linguistically diverse backgrounds. The lack of strategies and policies on how to design and implement virtual care to improve access to these services for this population was more noticeable during the pandemic.

They have very different cultural backgrounds and expectations on the way that healthcare is delivered. And I don't think we have fully unpacked what that looks like for those particular groups in our community.

Participant C1

#### Effectiveness Assessment for Postpandemic Use and Integration

Not all of the digital health technologies implemented during the pandemic were evaluated for sustainability and integration with workflow, as outlined by participant P1.

The formal evaluation hasn't happened, so there's no written evaluation of these services yet. But looking at the broader applicability to stretch it out beyond its original parameters has certainly taken off because of the success of how we've been able to run it currently.

Participant P1

The same participant emphasized that continuous evaluation and changes to IT infrastructure in health care settings are restricted due to financial impacts.

Some health care settings evaluated their virtual care services via the patient experience and patient outcome surveys in order to identify areas for improvement, as noted by participant P2. Moreover, participant C1 mentioned that the digital health technology evaluation requires a workforce to do research on it.

We need a workforce to do this and the barriers we have to do this are time and manpower. We don't have enough staff to be able to do the research that we need to do to evaluate this.

Participant C1

### Competency Requirements for the Future Nursing Workforce

This part of the interview reported participants’ expectations of new nursing graduates’ digital health capabilities and their ability to use technologies efficiently (Table 3).

**Table 3:** Participants’ expectations of nursing graduates’ digital health capabilities.

Participant group^a^	Expectations (participant ID^b^)
Group C	Students should learn about the rules and regulations of data security, privacy, and social media in using digital health. (C1 and C10) Students should be taught about nursing digital health capabilities before coming into practice. Use of academic electronic medical records should be a requirement in nursing programs. (C10) Nursing students should learn about digital health systems in more detail than only data entry (eg, data exchange, security, and analytics). (C11 and C12) Students should be taught about real-world digital health challenges in nursing in addition to theoretical concepts. (C11)
Group P	There is a need for investment in a digitally enabled nursing workforce, as they are the only providers in remote areas of Australia. (P1 and P2)
Group V	Universities can embed training content about analytics to foster critical thinking and curiosity among nurses about digital health technologies. (V1) Universities can provide simulated learning experiments for various digital health scenarios. (V1) The concept of a multidisciplinary approach should be embedded in digital health training to nursing students. They need to learn how to interact with internal and external stakeholders. (V1) Universities should foster digital health training to be responsive to the new generation of technologies. (V2)

^a^Group C included nurses in clinical settings, including chief nursing information officers, clinical educators, and nurses within health care settings who had experienced the use of digital health initiatives for patient care; Group P included nurse representatives in the government who contributed to policy making for digital health implementation in health care settings; and Group V included nurse representatives in digital health vendor companies that partnered with health care settings for digital health implementation.

^b^The participant IDs are clarified in Table 1.

Clinical nurses and clinical educator groups acknowledged that they do not have enough digital health literacy. However, this will not be the case for most upcoming nursing professionals as they are naturally technology literate owing to their generation being raised with digital technologies. They highlighted the need for practical skills in simulated training so that the future workforce will be able to apply their digital health knowledge and skills in practice. It is critical to invest in a digitally enabled nursing workforce during undergraduate education. This was highlighted by participant P2.

An investment in enabling that particular profession to be digitally enabled is important because it assists in dealing with the geographic divide. It allows organizations to have confidence that there's access to health care in a timely manner. For example, improved responses to emergencies and quick access to clinical data for diagnosis. It also allows us to understand the level of service delivery needs of that population when there's a public health emergency and or a burden of chronic.

Participant P2

The nurse representatives in the digital health market had similar expectations to those of the clinical nurse group, emphasizing training of detailed content of digital health and improvement of practical skills. A multidisciplinary approach in using digital health was outlined by participant V1 as a critical training need for nursing students.

We're in a space where we need to bridge the gap between the different professions that we're not just talking to other health professionals, we're talking to scientists across a very wide variety of topics and be able to transfer, to actually communicate the patient experience to a mathematician that this translates later into a solution that will be applied to the patient.

Participant V1

## Discussion

### Principal Findings

The pandemic brought an unprecedented shift in digital health adoption. Health care organizations began to implement various technologies to help increase the speed of clinical workflows. However, the interview findings showed that most of the clinical nurse participants did not have comprehensive knowledge of the functionality of digital health tools and applications used in their health care settings. They were also not comprehensively involved in digital health implementation processes to fully understand the facilitators and obstacles to efficient use of the technologies. The lack of systematic policies and procedures for digital health evaluation not only makes nurses passive users of the technologies but also prohibits a thorough understanding of sociotechnical issues that need improvements within the nursing workflow [29]. The fast-growing digital health market requires prompt changes in policies to adopt the technologies in health care.

All of the participants indicated that the pandemic interrupted the move from face-to-face care to virtual care, and many staff did not have formal training to cope with these changes. Moreover, most clinical nurses said that they received training from a colleague who had learned it previously instead of receiving formal training. The interviews identified a lack of leadership roles like chief nursing information/informatics officers who can be actively involved in digital health implementation. These professionals can have a pivotal role in coordinating the team and providing necessary training.

Nurses mainly used digital health technologies during the pandemic for virtual communication with patients and other professionals, remote monitoring of patients with either COVID-19 symptoms or chronic diseases, and training. A study by Isidori et al [9] found similar usage of digital health among nurses in a literature review with a 10-year timeframe that included studies conducted during the pandemic. Nurses experienced difficulties in nursing tasks and workflows when moving from one health care setting to another, where digital health maturity varies.

### Gaps in the Nursing Workforce’s Involvement in the Digital Health Pipeline

This study intended to explore the experiences of nurses involved in digital health design, development, implementation, use, evaluation, regulation, and policy making. The findings in Table 2 showed that the participants expressed sociotechnical challenges during the implementation and use of digital health technologies in nursing practice, with no indication of the design or development of such technologies requiring modification from their perspectives, which might not have been the core priorities for health care organizations during the pandemic. However, even before the pandemic, there was a lack of evaluation processes in health care settings, which, if conducted, would have revealed more detailed issues that can be considered by digital health technology vendors.

A study showed that devices designed and developed without nurses’ inputs negatively impacted nursing workflow and patient safety [36]. The lack of appropriate leadership and internal and external policies on digital health evaluation in nursing practice to provide insights for design or development improvement was reported in this study as a major barrier to nurses’ engagement in the effective use of these initiatives. Technology developers and implementers need to understand the complexity of care processes within the nursing practice. Nurses as end users have a vital role in facilitating a shared understanding. This highlights the need for the involvement of nurses in the design and development to capture their experiences and insights into how the technology will respond to their real-world practice needs [37]. This could be leveraged by a strong nursing leadership to shift the organizational culture and provide collaborative work to enable nurses to actively engage in the digital health pipeline and create a feedback loop to facilitate collaboration and provide opportunities to hear nurses’ concerns regarding technology usability [38,39]. Design, development, and implementation of digital health without any input from nurses would lead to inefficient workload and burnout that might lead to unsafe care delivery [40].

In general, among the groups of interviewees, the group of clinical nurses expressed the slightest awareness and involvement of digital health interventions in their health care settings. Their knowledge was limited to the technologies used in their department and not in other nursing practices within their care settings. Nurses might have the knowledge needed to improve the application of digital health technologies, but this research showed that they are not involved in the evaluation. Owing to the lack of an assessment, there is no input to return to the vendor for redesigning or improving the development or co-design strategies with nurses [36].

### Implications for Nursing Digital Health Competencies

The current nursing curricula lack comprehensive informatics and digital health content about all aspects of the digital health technology lifecycle, which prevents nurses from keeping up with the digital transformation [41,42]. This study showed that most of the clinical nursing groups perceived a lack of competency in digital health. The participants were asked about their expectations of digital health competencies ranging from basic skills, such as only awareness of digital health concepts or theoretical knowledge, to advanced skills, such as practical skills and evaluation. However, their responses were generic and without detailed specifications. Content beyond digital documentation was suggested to familiarize nurses further with the technical functionality and ability for interpretation. To ensure that the nurse workforce contributes to shaping the future of digital transformation, nursing education must embrace related content, and the nursing curricula should be updated to reflect contemporary nursing informatics practices [43].

### Comparison With Prior Work

Several qualitative studies have been published since the beginning of the pandemic, and they explored the impact of digital health implementation on nursing practice [29,44-46]. Some studies have discussed telehealth and virtual care interventions in general with less focus on nurses. This study is the first of its kind in Australia to investigate the sociotechnical issues in the digital health pipeline that impact nursing performance, and it involved 5 groups of nurses across the country.

### Limitations

The main limitation of this study was the lack of diversity in the geography of the participants from across the country. In addition, this research was conducted in the second year of a pandemic during a time of significant workforce shortage and high clinical demand. It is assumed that these factors influenced the ability to recruit nurse professionals for this study.

### Conclusions

The rise of digital health adoption and use in health care settings through the proliferation of various technologies is an essential shift brought on by the pandemic. This interview study explored the level of involvement of nurses and the sociotechnical challenges they face in the digital health pipeline. There is a lack of knowledge, engagement, policies, leadership, and training that has led to various challenges in nursing digital health practice. Future work can dive deeper into the sociotechnical issues for different technologies to provide insights for co-designing tools with nurses, which can meet their needs and provide safe and quality care, and the curricula can be revised to increase digital health competencies in the emerging nursing workforce.

## Abbreviations

EMR: electronic medical record

## Data Availability

The data underlying this article cannot be shared publicly owing to the privacy concerns of the individuals who participated in the study.
